# Laparoscopic trans-cystic common bile duct exploration and treatment of choledocholithiasis in a patient with Roux-en-Y reconstruction after gastrectomy: report of an emergency case

**DOI:** 10.1093/jscr/rjab144

**Published:** 2021-04-27

**Authors:** Marco Giacometti, Francesco Battafarano, Orazio Geraci, Sandro Zonta

**Affiliations:** Department of General Surgery, Ospedale San Biagio, ASL VCO, Domodossola, Italy

## Abstract

We present the case of choledocholithiasis with purulent cholangitis treated with laparoscopic approach in a patient with Roux-en-Y reconstruction after total gastrectomy. After cholangiography, the common bile duct was explored with trans-cystic choledochoscopy and the retained stone extracted under direct vision.

## INTRODUCTION

The presence of stones in common bile duct (CBD) is reported in 10–18% people undergoing laparoscopic cholecystectomy (LC) for symptomatic cholelithiasis [1], with percentage higher in patients admitted for acute cholangitis.

Procedures to clear CBD from obstructive gallstones include endoscopic retrograde cholangiopancreatography (ERCP) performed before, during or after LC and laparoscopic common bile duct exploration (LCBDE) during LC. To date, there is no demonstration of which method is the better choice, being both with pros and cons in terms of skills required, effectiveness, early and late complications. Moreover, endoscopy could be excluded when patient underwent gastric surgery because approaching the bile duct is challenging.

In our institution, we treat detected CBD stones with endoscopy (ERCP) before surgery (LC), in elective as in emergency setting.

We present the case of a patient with previous total gastrectomy, admitted with acute cholangitis from CBD lithiasis and treated with trans-cystic LCBDE and stone extraction.

## CASE REPORT

A 51-year-old male was admitted to our General Surgery Unit on March 2020, during the earlier weeks of severe acute respiratory syndrome coronavirus 2-related pandemic.

The patient had access to accident & emergency (A&E) department with abdominal pain, nausea and fever for the last 48 h. He had a history of hypertension, ulcerative colitis, cholelithiasis and previous laparoscopic total gastrectomy (2012) with D2 lymphadenectomy and Roux-en-Y reconstruction for a gastric adenocarcinoma (tumour, node, metastasis (TNM): G3 pT1 pN0 M0, no adjuvant chemotherapy—completed oncological follow-up).

**Figure 1:**
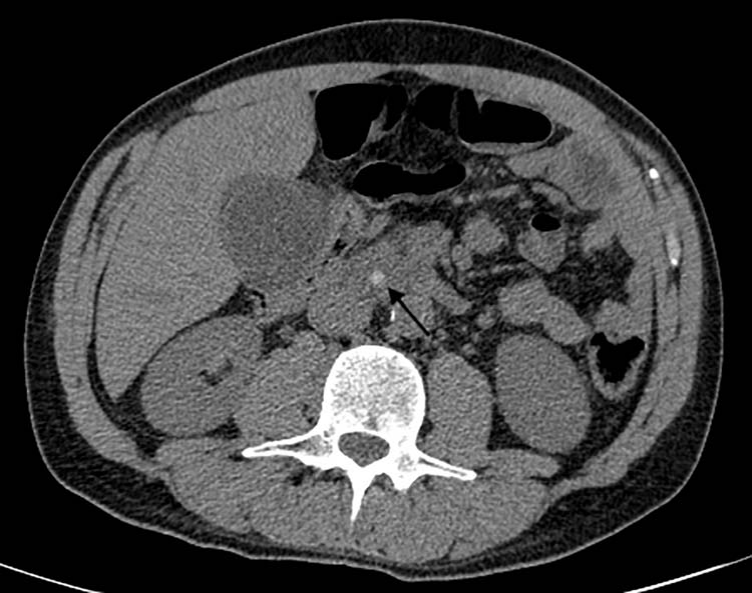
Computed tomography scan image showing stone in CBD.

**Figure 2:**
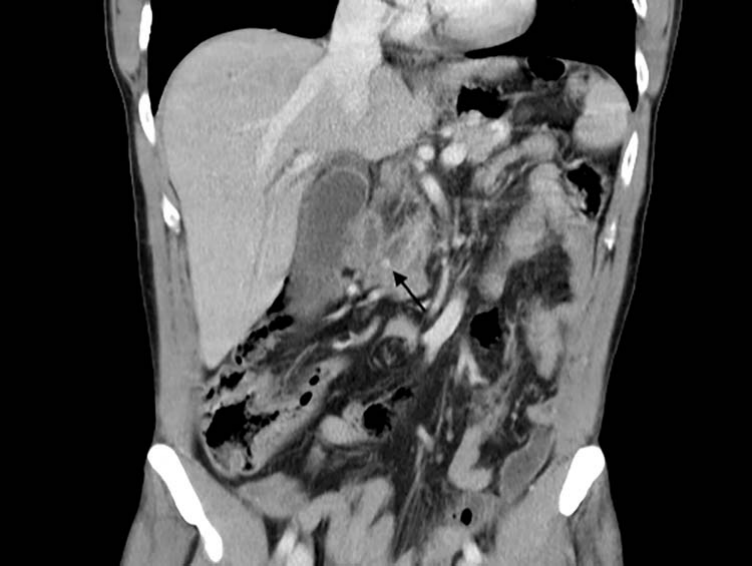
Computed tomography scan image showing stone in CBD.

Blood tests performed at admission demonstrated cholestasis and high transaminases. An abdomen computed tomography scan with intravenous contrast was performed, displaying a lithiasic cholecystitis and cholangitis, with a radio-opaque stone in the terminal tract of the CBD (Figs 1 and 2).

Patient was febrile, with tenderness at abdominal high right upper quadrant and signs of initial sepsis, not responding to broad-spectrum antibiotics. ERCP was not considered feasible due to previous gastric surgery and reconstruction. Also, trans-parieto-hepatic drainage was not an option because of absent dilatation of intrahepatic biliary ducts.

The morning after admission the patient underwent surgery, with an equipe (M.G., F.B., and O.G.) skilled in laparoscopic elective and emergency surgery and biliary surgery.

After pneumoperitoneum with Veress needle, four trocars were placed (12 mm, umbilical; 12 mm, epigastric; 5 mm, right upper quadrant; 5 mm, right flank) and a 30° camera was introduced. An empyema of gallbladder was detected, with dilatation of cystic duct and CBD. After closure of the infundibulum with a clip, cystic duct was incised with spillage of bile and pus.

**Figure 3:**
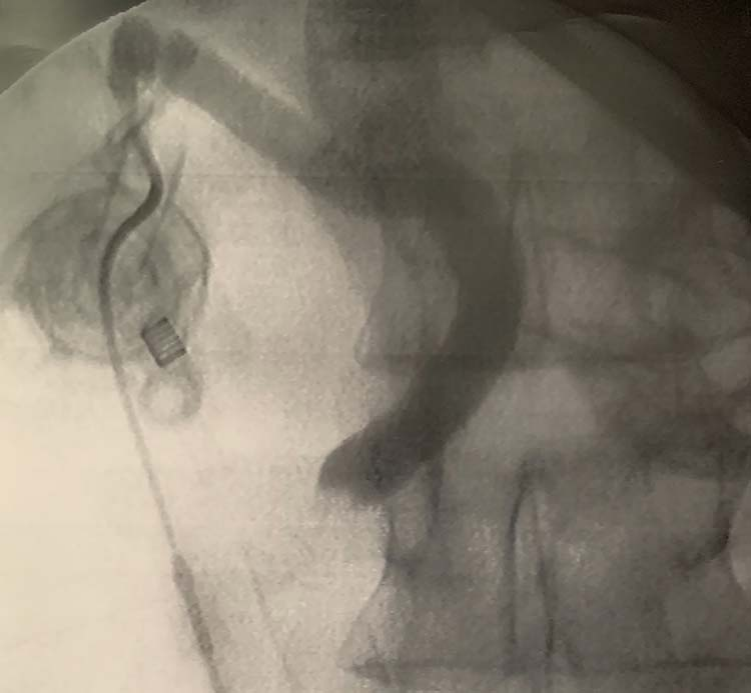
Trans-cystic cholangiography demonstrating prepapillary lithiasis.

A trans-cystic cholangiography was then performed, using a 5.0 Fr COOK^®^ Medical ureteral catheter inserted through epigastrium. The augmented caliber of CBD and the presence of prepapillary lithiasis were confirmed. No contrast could pass into the duodenum (Fig. 3).

Another 5 mm epigastric trocar was placed and cystic duct was dilated with silicone Bougies. Because of unavailable laparoscopic choledochoscope, a 3.1 mm Innovex^®^ flexible ureterorenoscope was employed to perform a trans-cystic LCBDE.

**Figure 4:**
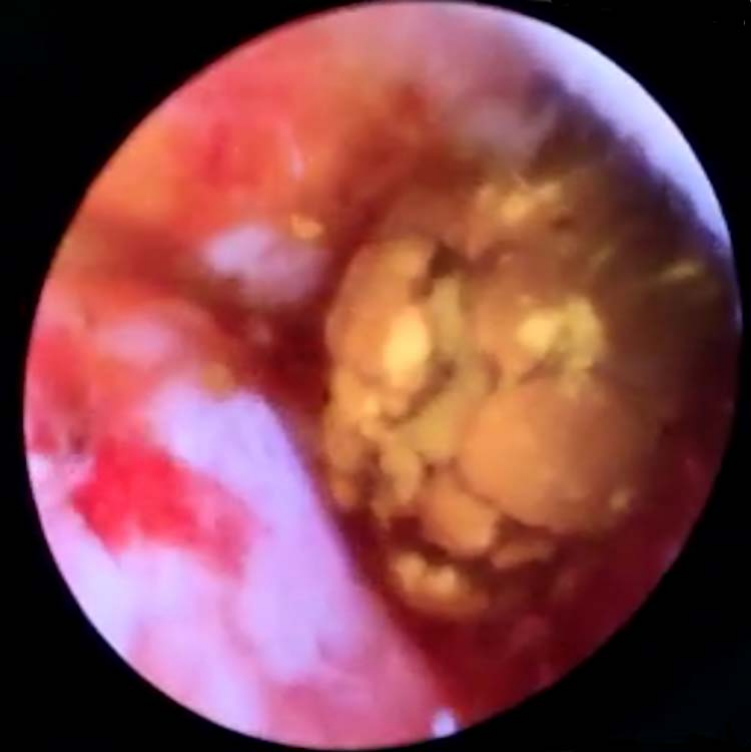
Choledochoscopic vision of CBD-retained stone.

**Figure 5:**
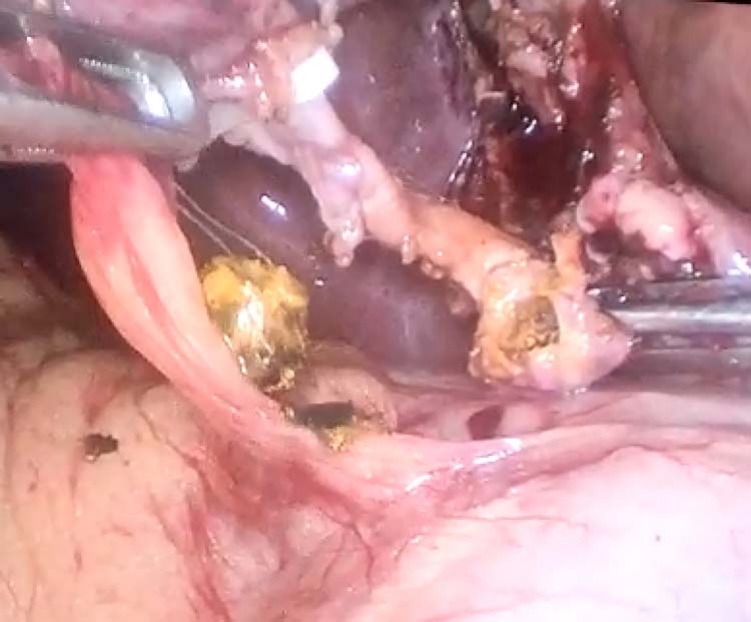
Trans-cystic stone extraction with Dormia basket.

**Figure 6:**
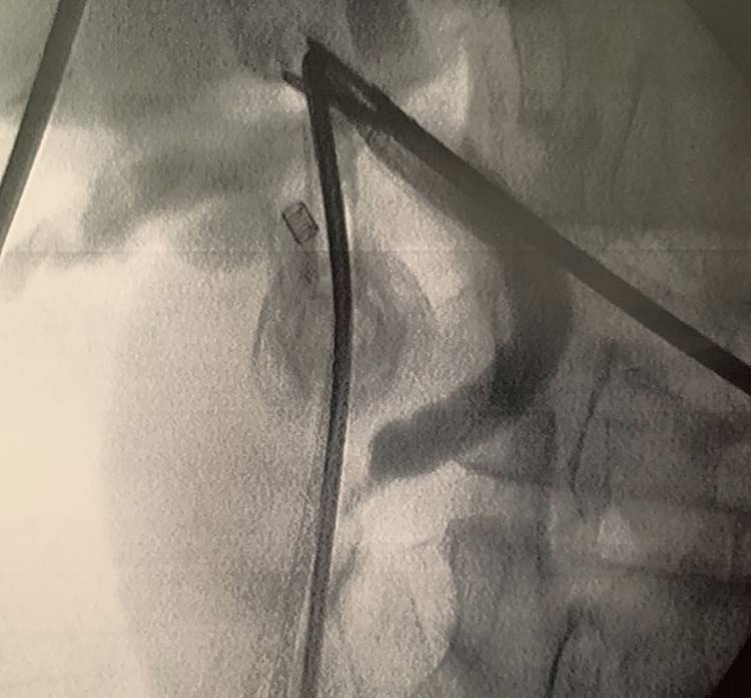
Postprocedure cholangiography.

In context of purulent cholangitis, a 10 mm stone was reached (Fig. 4) and extracted with Dormia basket under direct vision (Fig. 5). Furthermore, control choledochoscopy and cholangiography did not show any other stones in the CBD and contrast easily passed into the duodenum (Fig. 6).

Cystic duct was then closed with Hem-o-Lock^®^ clips and the operation ended with cholecystectomy and extraction with endo-bag. A single Jackson–Pratt drainage was placed under the liver.

Postsurgical course was almost uneventful, with only minor complications (Clavien–Dindo I), and the patient was discharged at home on 10th postoperative day after completing antimicrobial therapy. He was asymptomatic and with normal blood tests at 1-, 3- and 6-month follow-up.

## DISCUSSION

Laparoscopic trans-cystic choledochoscopy is described in literature since the early 90s [2, 3], and LCBDE during cholecystectomy has been recently stated as safe and effective by a meta-analysis [4], even if performed thorough choledochotomy, which is a procedure definitely more invasive than trans-cystic approach.

Recently, Japanese surgeons demonstrated a dual CBD examination with trans-cystic choledochoscopy and cholangiography during LC, with trans-cystic removal of CBD stones, is safe and feasible and reduces invasive treatments, complications of ERCP, days of hospital stay and medical costs [5]. However, acute patients were excluded from analysis.

The laparoscopic trans-cystic CBD exploration has been defined safe and effective also in emergency condition [6] with intraoperative cholangiogram. CBD detected stones, however, were removed with a trans-cystic Dormia basket and not under direct choledochoscopy view. Another paper [7] assessed emergency CBD exploration as safe and effective in elderly patients with complicated acute cholangitis.

Many different procedures of LCBDE are described in literature, divided into two main groups: through choledochotomy or trans-cystic. In our opinion, the latter is the best option when practically feasible, avoiding injuries of CBD and possible biliary leakage or late CBD strictures. Moreover, when performed under choledochoscopy direct vision, trans-cystic approach is safer even in particular cases. For example, LC + LCBDE has been described as successfully performed on a pregnant patient with cholecystitis and choledocholithiasis [8] and in a patient with gallbladder agenesis [9].

The risk of cholelithiasis after total gastrectomy for gastric cancer has been widely reported in literature, with possible CBD stones, which can be hardly removed through ERCP. A recent paper [10] has described a one-stage fluoroscopy-guided laparoscopic trans-cystic papillary balloon dilation, pressure washing and LC in patients with cholecystocholedocholithiasis after gastrectomy.

To our knowledge, to date, our is the first published case of purulent cholangitis with choledocholithiasis in a patient with Roux-en-Y reconstruction after gastrectomy, treated in an emergency setting with completely laparoscopic approach and with trans-cystic removal under choledochoscopic direct vision.

LCBDE and stone extraction require very good laparoscopic surgery skills, appropriate operative room setting and proper surgical instruments for CBD endoscopy. For this reason, ERCP + LC is still preferred in many institutions because it is easier to apply in the majority of cases, even though LCBDE has been demonstrated as safe and effective, with much less complications if performed with trans-cystic direct vision. We think trans-cystic LCBDE is an option that should be offered in centers performing high-level minimally invasive surgery, at least in cases, which cannot be treated with ERCP + LC.

## CONFLICT OF INTEREST STATEMENT

None declared.

## FUNDING

None.
